# The Jurassic magmatism of the Demerara Plateau (offshore French Guiana) as a remnant of the Sierra Leone hotspot during the Atlantic rifting

**DOI:** 10.1038/s41598-020-64333-5

**Published:** 2020-05-04

**Authors:** Christophe Basile, Igor Girault, Jean-Louis Paquette, Arnaud Agranier, Lies Loncke, Arnauld Heuret, Ewald Poetisi

**Affiliations:** 1 Univ. Grenoble Alpes, Univ. Savoie Mont Blanc, CNRS, IRD, IFSTTAR, ISTerre, 38000 Grenoble, France; 2 Université Clermont Auvergne, CNRS, IRD, OPGC, Laboratoire Magmas et Volcans, F-63000 10 Clermont-Ferrand, France; 3 Laboratoire Géosciences Océan (UMR CNRS 6538), Université de Bretagne Occidentale & Institut Universitaire Européen de la Mer, Place Nicolas Copernic, 29280 Plouzané, France; 4 Université de Perpignan, CEFREM – UMR 5110, 66860 Perpignan, France; 5 Université de Guyane, Géosciences Montpellier, 97300 Cayenne, France; 6 Anton de Kom University of Suriname, Paramaribo, SA Suriname; 7 Present Address: IDEES (UMR 6266 CNRS), Université de Rouen, Rouen, France

**Keywords:** Biogeochemistry, Solid Earth sciences

## Abstract

We report the discovery of 173.4 Ma hotspot-related magmatic rocks in the basement of the Demerara Plateau, offshore French Guiana and Suriname. According to plate reconstructions, a single hotspot may be responsible for the magmatic formation of (1) both the Demerara Plateau (between 180 and 170 Ma) and the Guinea Plateau (circa 165 Ma) during the end of the Jurassic rifting of the Central Atlantic; (2) both Sierra Leone and Ceara Rises (mainly from 76 to 68 Ma) during the upper Cretaceous oceanic spreading of the Equatorial Atlantic ocean; (3) the Bathymetrists seamount chain since the upper Cretaceous. The present-day location of the inferred Sierra Leone hotspot should be 100 km west of the Knipovich Seamount.

## Introduction

**Figure 1:**
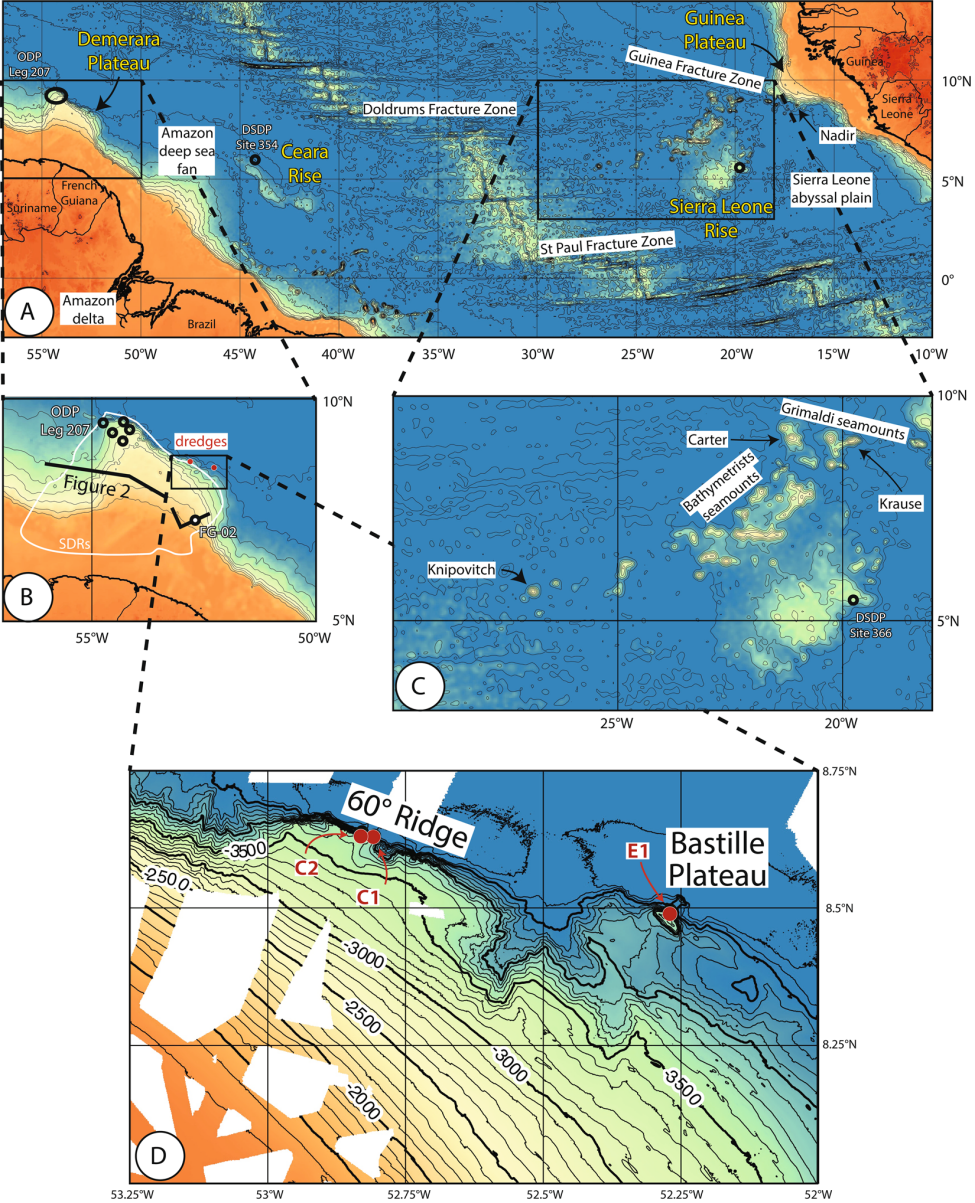
(**A**) topography and toponomy of the northern part of the Equatorial Atlantic. (**B**) Demerara Plateau; (**C**) Sierra Leone Rise and Bathymetrists seamounts. Same scale for (**B,C**). Isobaths every 500 m for (**A–C**) (Bathymetric data from Geomapapp.org v. 3.6.10). 1D: Location (red dots) of DRADEM dredges that recovered magmatic rocks. Bathymetry (depths in meters) from GUYAPLAC^10^, IGUANES^39^ and DRADEM^6^ cruises.

In the northern part of the Equatorial Atlantic, between the St Paul and Doldrums fracture zones, two pairs of submarine plateaus form outstanding reliefs (Fig. 1A):

- The Demerara and Guinea marginal plateaus, which extend seaward the continental shelf and slope offshore Suriname and French Guiana on the American side, and offshore Guinea and Guinea-Bissau on the African side;

- The Ceara and Sierra Leone rises, high standing on the abyssal plain on each side of the mid-Atlantic ridge.

The Sierra Leone and Ceara rises constitute an oceanic Large Igneous Province (LIP), formed during the upper Cretaceous at or close to the Atlantic mid-oceanic ridge, and split in two parts during the subsequent oceanic spreading^1^. The Sierra Leone hotspot^2^ has been inferred from these submarine reliefs, but its present-day location is elusive, as it has not been related to active or recent aerial or submarine volcanism.

**Figure 2:**
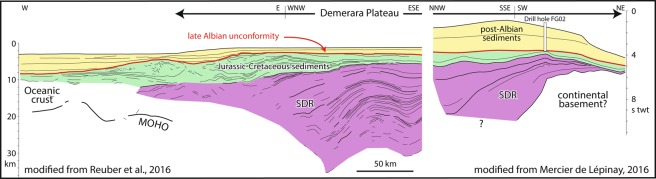
Schematic seismic lines across the Demerara Plateau, modified from Reuber *et al*.^4^ and Mercier de Lépinay^3^. Westward, vertical scale is in km, eastward in seconds two-way travel time. The lines are located in Fig. 1B.

The Demerara and Guinea marginal plateaus also represent conjugated structures, which were initially connected, before being pulled apart during the lower Cretaceous by the opening of the Equatorial Atlantic Ocean. Recent studies^3,4^ showed that the basement below these two marginal plateaus consists of Seaward Dipping Reflectors (SDRs), up to 20 km-thick in Demerara (Fig. 2). Those thick SDR packages suggest that the two plateaus may have formed during continental rifting as magmatic divergent margins, and may be part of a Large Igneous Province^5^ (approximate surface 0.15 Mkm^2^; volume 0.6 Mkm^3^ for Demerara only, calculated from seismic sections published by Reuber *et al*.^4^).

In 2016 the DRADEM oceanographic cruise^6^ dredged the northern slope of the Demerara Plateau in order to sample the outcropping basement. In this paper we present geochemical and dating results from two sites where magmatic rocks were recovered, and we discuss if both marginal and oceanic plateaus can be related to a single hotspot in this part of the Atlantic Ocean.

### Geological setting

South of the Guinean Plateau, and East of the Demerara Plateau, the northern Equatorial Atlantic is an oceanic basin that resulted from continental rifting during the lower Cretaceous (Barremian-Aptian), and oceanic spreading since Late Aptian times^7^. This rift and the subsequent oceanic spreading axis connected the South Atlantic Ocean (lower Cretaceous in age) with the Central Atlantic, where oceanic spreading started during lower to middle Jurassic^8^. Consequently, the western edges of the Guinea and Demerara plateaus are Jurassic continental divergent margins, while the South Guinea Plateau, North and East Demerara Plateau represent Cretaceous continental margins. Furthermore, North Demerara and South Guinea plateaus are conjugated transform margins^9,10^, and thus both plateaus can be defined as transform marginal plateaus^11^. In both plateaus, the uppermost sedimentary section has been drilled (ODP Leg 207^12^, FG02 drillhole^3,13^; Fig. 1B), but the underlying basement was not sampled up until the DRADEM cruise.

The Ceara Rise is an elongated plateau, 700 km long and less than 200 km wide (Fig. 1A). Its surface is gently dipping to the north, and its southern edge is a steep slope acting as a dam that restrains the spreading of the Amazon deep sea fan. The Sierra Leone Rise is 700 km × 350 km in size, gently dipping to the northwest, with a 2 km-high steep eastern slope above the Sierra Leone abyssal plain (Fig. 1C). Geological data are very sparse in both areas. Deep Sea Drilling Project (DSDP) sites 354 (Fig. 1A) and 366 (Fig. 1C) drilled Ceara and Sierra Leone rises, respectively. The oldest recovered sediments are Maastrichtian in age in both drill holes^14^. These sediments overlie basalts at sites 354, but older interlayered sediments were suggested from local high coring rates, making a common 80 Ma age likely for the initiation of magmatic accretion in both rises^14^. Jones *et al*.^15^ showed that abnormally thick oceanic crust (up to 17 km) underlies the Sierra Leone Rise.

Several groups of seamounts follow the northwest edge of the Sierra Leone Rise (Fig. 1C). They are aligned either in E-W or NE-SW directions. The Grimaldi seamounts follow the E-W trending Guinea Fracture Zone (Fig. 1A), which ends eastward with the transform margin south of the Guinea Plateau. Carter, Krause and Nadir seamounts belong to this chain (Fig. 1A,C). They were emplaced at 58, 54 and 59 Ma, respectively^16–18^. South of the Guinea Fracture Zone, the Bathymetrists seamounts consist of two parallel chains trending NE-SW, ending at 7°N with an E-W trending chain of seamounts (Fig. 1C). This E-W chain has been sampled by dredging, demonstrating that the seamounts are magmatic in origin, hotspot related, and capped by a carbonate platform that developed at sea-level during middle Eocene times^19^. The westernmost extremity of the Bathymetrists chain is the isolated Knipovitch seamount, with a horizontal quadrangular summit approximately 12 km x 6 km in size at less than 600 m water depth^20^. Maher *et al*.^21^ interpreted the Bathymetrists chain as a hotspot track, initiated at the Carter seamount and presently centered 160 km south of Knipovitch seamount, where it has no bathymetric expression.

### Dredging sites

The DRADEM cruise^6^ dredged six sites along the northern slope of the Demerara Plateau. In two sites (Fig. 1D), a total of three dredges recovered the magmatic rocks on which we focus in this paper. In the other sites, the dredges recovered only sedimentary rocks.

Two dredges were operated on the northern slope of a bathymetric structure called the 60° Ridge at the northeastern edge of the Demerara plateau (Fig. 1D). This WNW-ESE-trending asymmetric ridge rises up to 3400 m deep. It stands up to 200 m above the edge of the Demerara plateau to the south, and 1200 m above the abyssal plain to the south. The average northern slope is 40°, locally 60° towards the crest of the ridge. This steepness is supposed to be inherited from the strike-slip deformation that occurred during the Cretaceous formation of this transform margin. The deep dredge (C1) was operated from 4540 to 4150 m depths, the shallower one (C2) from 4250 m to the crest of the ridge at 3400 m depths.

One dredge was operated on the northern slope of a small plateau (called Bastille Plateau) at the northeast corner of the Demerara Plateau (Fig. 1D). Bastille Plateau presents an almost flat surface at 3700 m depth, standing 300 to 400 m above the deep part of the Demarara plateau (lower plateau^10^), and 1000 m above the abyssal plain. The Bastille Plateau is elongated in a NW-SE direction, which is oblique relative to the regional trend of the northern edge of the Demerara plateau. Dredge E1 was operated on the northern slope of the Bastille plateau, from 4400 m up to 3740 m depth.

## Results

### Petrography

**Figure 3:**
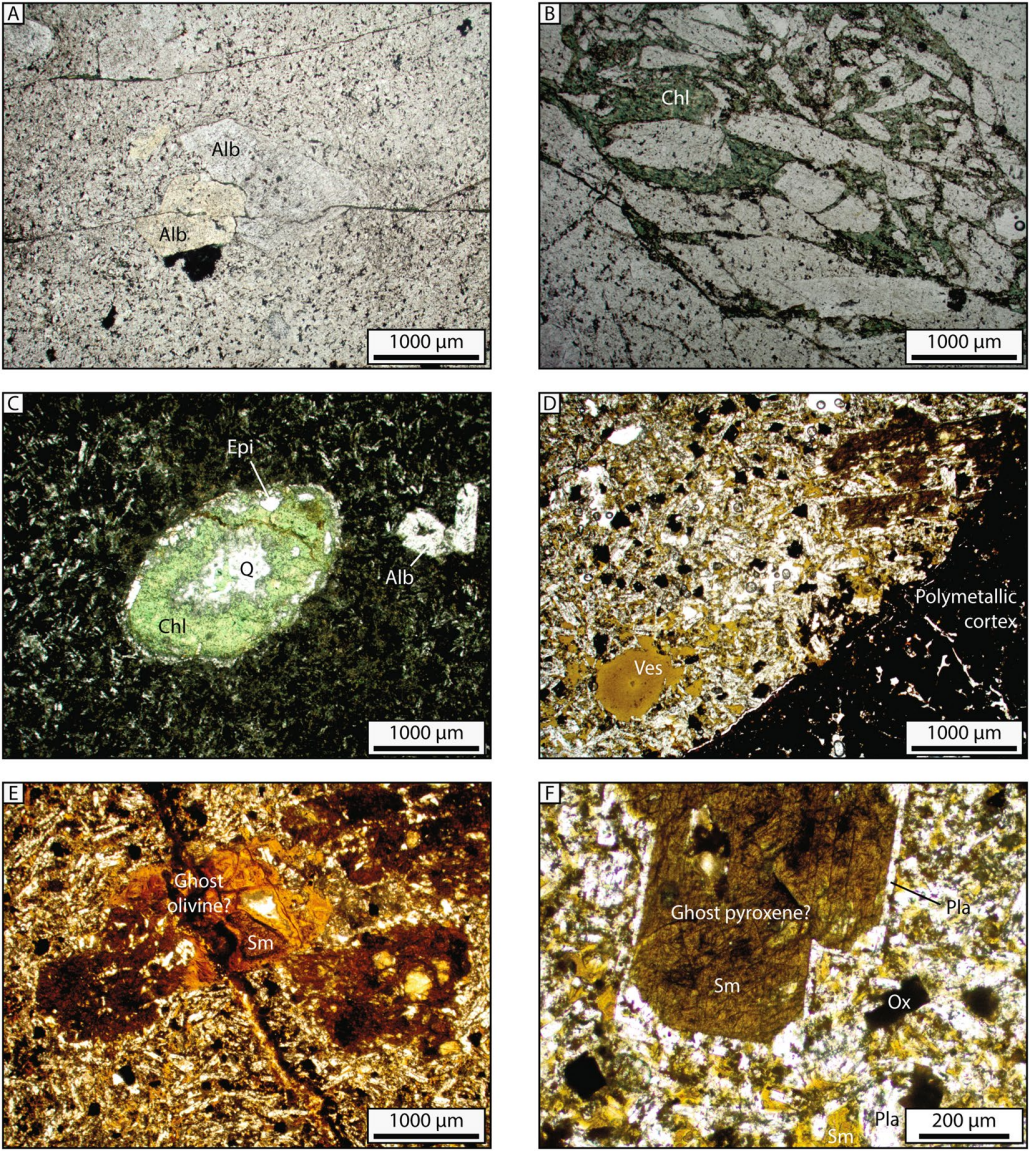
Thin sections of magmatic samples, in plane-polarized light. A and B from C1 dredge (samples C1-3 and C1-4); C from C2 dredge; D to F from E1 dredge (sample E1-9B). Alb: Albite; Chl: Chlorite; Epi: Epidote; Q: Quartz; Ves: Vesicle filled with palagonite, Sm: Smectite; Pla: Plagioclase; Ox: Oxide.

The three dredges recovered different types of magmatic rocks (Fig. 3):

Dredge C1 (deep part of 60° Ridge) recovered four pieces with similar lithologies. They present a porphyritic texture, with albite phenocrysts in a quartz and feldspar groundmass (Fig. 3A). Epidote and chlorite veins cut the groundmass (Fig. 3B).

Dredge C2 (shallow part of 60° Ridge) recovered a single block of volcanic rock with microlithic texture, with very few albite phenocrysts in actinote, plagioclase and chlorite mesosthasis (Fig. 3C). Millimetric amygdales are filled either with quartz crystals, or by concentric layers of epidote, quartz and chlorite (from the side to the center) (Fig. 3C). All amygdales flatten along parallel surfaces.

From their textures, these rocks are interpreted as emplaced either in the underground as magmatic stock or dykes (C1), or close to the surface (C2) where magmatic degassing can produce vacuoles, flatten during the compaction of the lava, then filled by secondary minerals. In both cases, epidote and chlorite point out greenschist metamorphic facies, likely to have occurred during post-crystallization cooling for C2, possibly during later deformation for C1. No macroscopic or microscopic sea water alterations were observed in relation to the outside surface of C1 and C2 samples.

Dredge E1 (Bastille Plateau) recovered numerous clasts of magmatic rocks, homogeneous in macroscopic examination. They present a porphyritic texture, with numerous feldspar phenocrysts (Fig. 3D–F). These magmatic rocks were strongly altered, and smectite represents 15 to 30% of the groundmass. Secondary minerals (including smectite) fill vesicles and the spaces in the feldspar lattice (Fig. 3D), suggesting the formation of palagonite from low temperature alteration of volcanic glass in contact with water. In places, one can observe some pseudomorphs of probably former pyroxene (Fig. 3F) and olivine crystals (Fig. 3E).

### Geochemistry

Samples cover a wide range of compositions, from basaltic (C2) to rhyolitic (C1) for the 60° Ridge, and trachy-basalts for the Bastille Plateau (E1). SiO_2_ concentrations range from 45 to 73 wt%, MgO = 0.2–5.3 wt% and (Na_2_O + K_2_O) = 4.1–7.8 wt% (Table S1). Loss On Ignition (LOI) is only 0.3% for rhyolite (DRA-C1, Table S1), 2.1% for basalts (DRA-C2, Table S1), where it is probably related to epidote and chlorite that fill the vacuoles. LOI ranges from 3 to 7% for the intermediate volcanic rocks (DRA-E, Table S1), and attests to their altered nature. At least for these intermediate rocks, it is likely that major elements have somehow been modified by interactions with meteoric or seawater fluids (e.g. SiO_2_ leaching, attested by the negative correlation of SiO_2_ with the LOI: Fig. S1), and classic magmatic systematics (for example total of alkali vs SiO_2_) should thus be considered with extreme care. The Ti contents are high, being 3% in the basalt from 60° Ridge, from 2.7 to 4.1% (3.5% in average) in the altered trachy-basalts from Bastille Plateau (Table S1).

**Figure 4:**
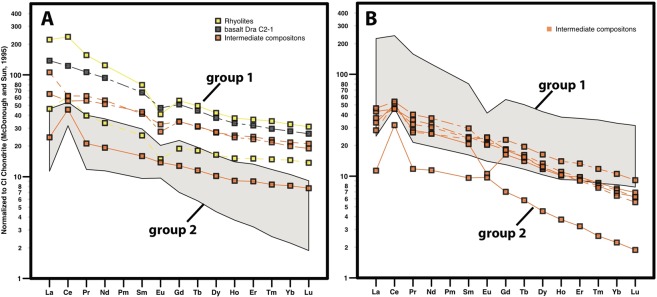
Rare earth element patterns normalized to CI Chondrite^60^. (**A,B**) Samples can be divided into two groups (1 and 2) of parallel spectra (likely co-genetic). Symbols discriminate samples considering their major element compositions with rhyolites-like (SiO_2_ > 70%, yellow squares: C1 samples), basaltic-like (gray square: C2 sample), and intermediate compositions (orange squares: E1 samples).

**Figure 5:**
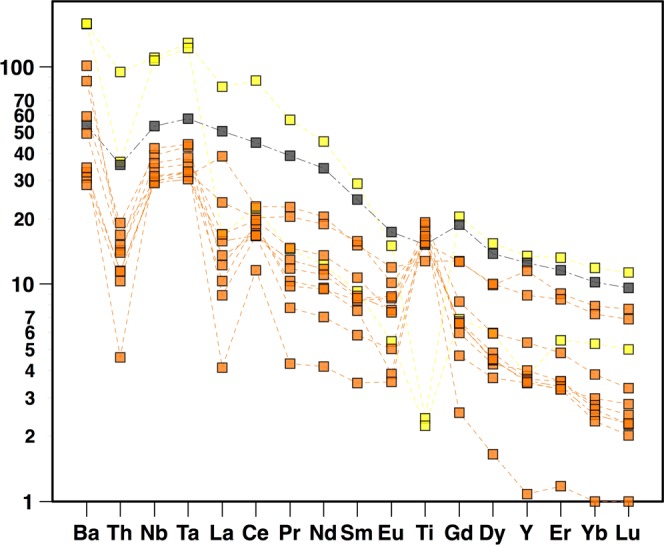
Trace elements normalized to Primitive Mantle^60^. Same symbols as in Fig. 4.

Trace elements also display a wide range of compositions (1 to 100 times the primitive mantle) possibly induced by various amounts of interactions with seawater. Nevertheless, rare earth element (REE) signatures (least likely fractionated by weathering processes) can be divided into two groups of almost parallel patterns (Fig. 4), suggesting a possible co-genetic link between two series of rocks. Cerium positive anomalies (Ce/Ce* = 1–2.7 with Ce* = (La+Pr)_N_/2 × (Ce)_chondrite_) (Fig. 4), could be accounted for by seawater interactions under oxidative conditions (Ce^4+^ being less mobile in fluids than Ce^3+^ and other REE), while Eu negative anomalies can be explained by plagioclase fractionation during magmatic evolution. On an extended trace element spider-diagram (Fig. 5), patterns are mostly dominated by these Ce anomalies, together with Ti, Ta and Nb enrichments. These high field strength element (HFSE) enrichments may also be related to selective leaching processes that may be responsible for the general depletion in all trace elements except for Ce^4+^ and HFSE. This hypothesis is supported by the correlation existing between Ce and Nb positive anomalies (quantified by Ce/Ce* and Nb/La ratios respectively) (Fig. S2); however, the extent of the HFSE positive anomaly being approximately three times higher than the Ce anomalies, it is likely that a pristine (i.e.: magmatic) enrichment in these elements was present before alteration.

All samples have mostly parallel trace element patterns, with light Rare Earth Elements (REE) enriched compared to heavy REE ((La/Yb)_N_ = 3–6) and positive Ti, Ta and Nb (“TITAN”) anomalies (Fig. 5). These patterns are common in Oceanic Island Basalts (OIB). Even if TITAN anomalies may be accounted for by partial melting, subsequent differentiations and crustal assimilations rather than deep source compositions^22^, they may still be indicative of a hotspot origin for the parental magma.

### Dating

**Figure 6:**
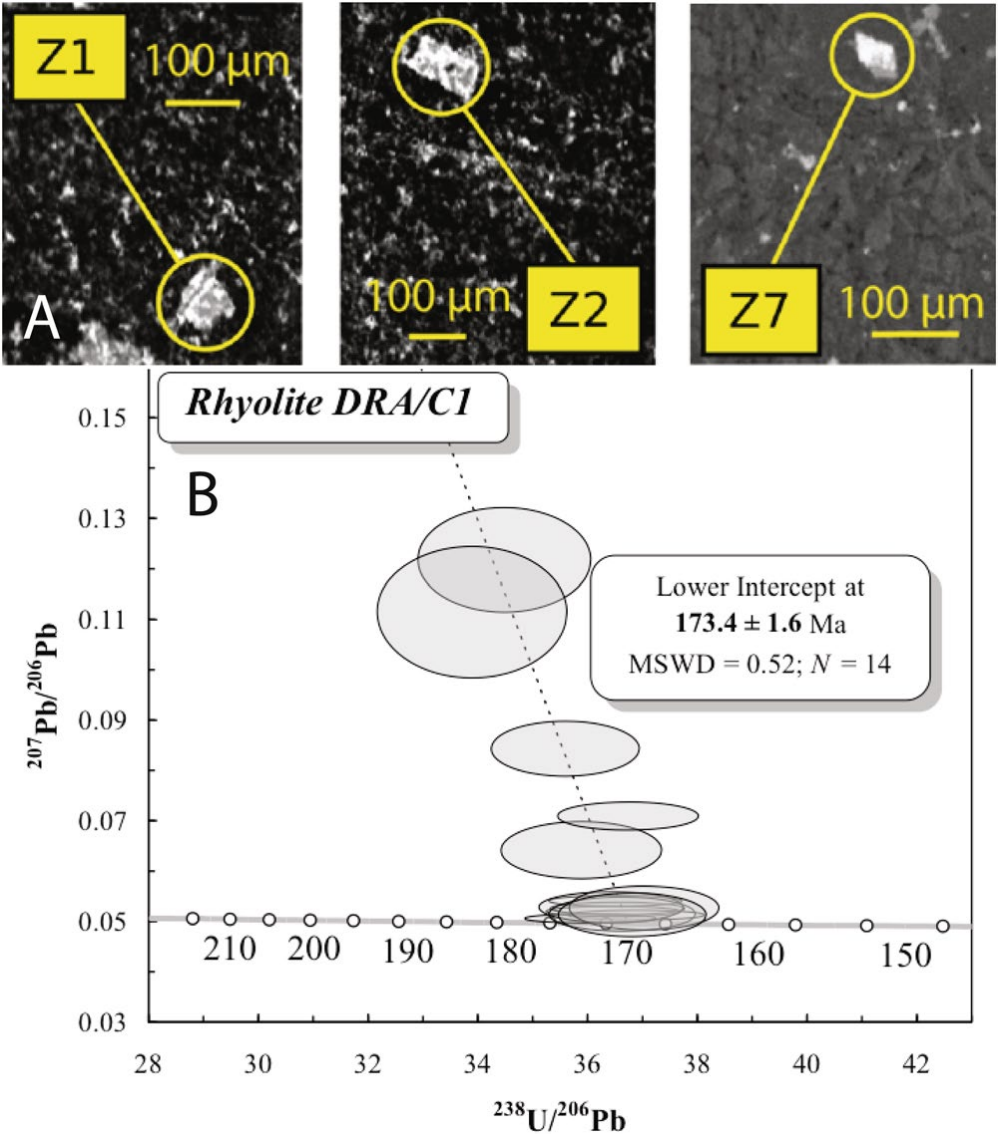
U/Pb dating of zircons. (**A**) Scanning Electron Microscope images of zircons in thin sections from samples DRA-C1-1 (Z1 and Z2) and DRA-C1-2 (Z7). (**B**) Tera-Wasserburg diagram of the DRA-C1 zircons.

We identified zircons in C1 rhyolites using a scanning electron microscope. They are less than 30 µm-long euhedral crystals, characterized by angular edges (Fig. 6A) and devoid of zonation when observed by cathodoluminescence. Fourteen 20 µm-wide laser ablation spots^23,24^ were performed on five zircon crystals in order to measure U/Pb isotopic compositions. They yield a lower intercept age of 173.4 ± 1.6 Ma (Fig. 6B and Table S2). This is interpreted as the crystallization age of magmatic zircons in the rhyolite, given the high Th/U ratios ranging from 0.5 to 1.3, and the absence of zonation.

### Kinematic reconstruction

The thick SDR packages observed on both the Demerara and Guinea plateaus suggest that these two plateaus constituted divergent magmatic margins during the Jurassic opening of the central Atlantic, and were likely related to a mantle plume event at that time^25^. Moreover, the OIB-like geochemical properties of the volcanic rocks sampled in this area also point toward a hotspot connection. Consequently, we propose to further investigate this hypothesis, by searching for a secular hotspot track. The kinematic reconstruction we used is based on the global plate motion model proposed by Müller *et al*.^26^. This model is anchored to a fixed reference, such as the spin axis. It uses a hybrid absolute reference frame, based on a moving hotspot model^27^ for the last 70 Ma, and a true polar wander model^28^ from 105 to 230 Ma, including a longitudinal shift of 10^◦^ from 100 to 230 Ma. From 70 to 105 Ma, Müller *et al*.^26^ introduced a transition from one reference frame to the other, in order to avoid unlikely plate displacements that may be inferred from a sudden change of reference frames.

**Figure 7:**
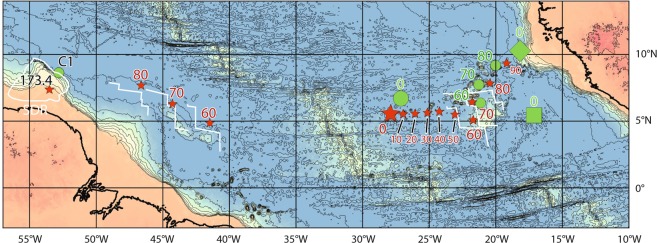
Reconstructed track of the Sierra Leone hotspot. The green dots and red stars represent the track of a hotspot located on the C1 dredge or below Demerara’s SDRs at 173.4 Ma, respectively, using the kinematic model of Müller *et al*.^26^; the large star and large dot being the expected present-day locations. Green square and diamond represent the present-day location of the hotspot centered on the C1 dredge at 173.4 Ma, using the kinematic models from Seton *et al*.^47^, and Matthews *et al*.^48^, respectively. White lines represent the oceanic spreading axis closer than 250 km to the hotspot (red star) at 60, 70 and 80 Ma (see also Fig. 8). Because the hotspot is close to the spreading axis from 80 to 60 Ma (less than 100 km), its track has been reported on both Africa and South America plates. The extension of SDRs below Demerara Plateau is from Reuber *et al*.^4^ and Mercier de Lépinay^3^. All ages are in Ma. Bathymetric data from ETOPO2 (ngdc.noaa.gov/mgg/global/etopo2.html) displayed using Qgis v. 2.14–3.

**Figure 8:**
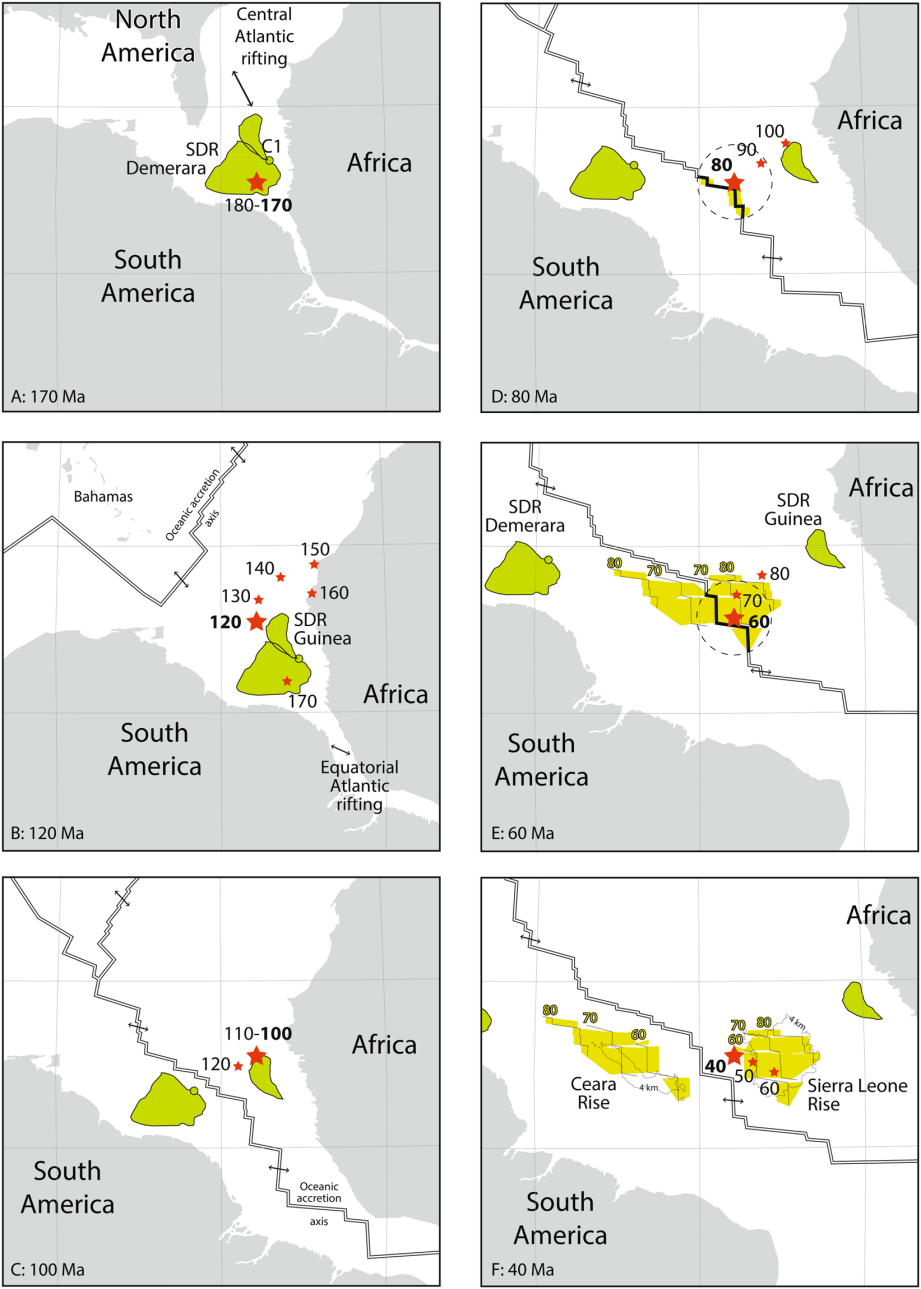
Track of a hotspot initially located below Demerara SDR’s at 173.4 Ma (red stars), using the kinematic model of Müller *et al*.^26^. The South America and Africa plates are almost immobile relatively to the hotspot from 180 to 170 Ma (**A**), during the supposed formation of the Demerara SDRs. Guinea SDRs may have formed either coeval with Demerara SDRs (**A**) or circa 165 Ma during the southward drift of the Africa plate above the hotspot (**B**). The Bahamas (**B)** may represent the track of the hotspot on the North America plate from 170 to 155 Ma^4^. The hotspot is closer than 100 km to the spreading axis from 82 to 55 Ma, with the light green surface representing the oceanic crust formed within 250 km from the hotspot (dotted circle **D,E**) in the same time span (thin lines in **E,F** are isochrones for 80, 70 and 60 Ma). In (**F**), the present-day 4 km isobath is superimposed for both Sierra Leone and Ceara Rises. Drawn from results using GPlates 2.0.0.

Based on the assumption that hotspots are fixed in reference to the rotation axis of the Earth, we investigated the track of a hotspot that was located beneath the Demerara Plateau 173.4 Ma ago. Using the location of C1 dredge as the center of the hotspot at 173.4 Ma, the model of Müller *et al*.^26^ reconstructs the hotspot close (less than 300 km) to the mid-Atlantic ridge from 80 to 60 Ma, at the expected time for the formation of the Ceara and Sierra Leone rises, and a present-day position north of the western end of the Bathymetrists chain (Fig. 7). The best fit with both Ceara – Sierra Leone rises and the Bathymetrists chain (Figs. 7 and 8) is provided moving the center of the hotspot 150 km SW of C1 dredge, close to the center of the SDRs mapped by Reuber *et al*.^4^ and Mercier de Lépinay^3^. Assuming a fixed hotspot at this location, the forward displacement of the moving plates fits with the following evidences:

- The thickness (up to 20 km, Fig. 2) of SDRs below the Demerara Plateau can result from the fixed location of the American plate above the hotspot from 180 to 170 Ma (Fig. 8A), suggesting that the magmatism may have occurred before the emplacement of the sample C1.

- The SDRs of the Guinean Plateau^3^: prior to the opening of the Equatorial Atlantic, this part of the Guinean Plateau was adjacent to the Demerara Plateau (Fig. 8A,B). Unfortunately, these SDRs have not been sampled or dated, although Mercier de Lépinay^3^ proposed from seismic lines a Jurassic age, older to contemporaneous with the onset of oceanic spreading west of the Plateau. The SDR of Guinea may be coeval with the SDRs of Demerara (Fig. 8A), or may be formed slightly later, when the Guinean plateau moved above the hotspot from 167 to 165 Ma (Fig. 8B).

- The Ceara and Sierra Leone rises: from 82 to 55 Ma, the postulated hotspot was located less than 100 km from the mid-Atlantic ridge axis, and at the ridge axis from 76 to 68 Ma (Fig. 8D,E). During this time span, and as postulated since Kumar^1^, the hotspot may have thickened the oceanic crust during its spreading. Since 55 Ma ago, the ridge axis moved away from the hotspot and the oceanic spreading pulled apart the two oceanic plateaus. The part of the spreading axis being closest than 250 km to the hotspot location from 82 to 55 Ma is in good agreement with both Ceara and Sierra Leone shapes (Figs. 7 and 8F). North of Brazil, the 80 Ma isochron (Figs. 7 and 8F) fits with the abnormally thick oceanic crust^29^, which may represent the westernmost part of the Ceara Rise hidden by the Amazon deep sea fan. The difference in shape (more circular for Sierra Leone, elongated for Ceara) can be explained by the difference in displacement of the lithospheric plates relatively to the hotspot, the South America plate moving faster than the African one.

- The Bathymetrists chain may represent the hotspot trail. In the model of Müller *et al*.^26^, the track of the hotspot from 90 to 70 Ma follows the northern NE-SW trending part of the Bathymetrists chain (Fig. 8D,E), while it follows the southern E-W trending chain from 50 Ma to the present day (Fig. 7). The Knipovitch seamount may represent the position of the hotspot 10 Ma ago. The present-day position may be 100 km west of the Knipovitch seamount.

- In this reconstruction, the formation of the volcanoes from the Guinea Fracture Zone (Carter, Krause, Nadir) is not directly related to the hotspot, being> 500 km far from it during their formation (54–59 Ma).

## Discussion

The magmatic rocks dredged during the DRADEM cruise represent a previously unknown magmatic event in this part of South America, both by their location and mid-Jurassic age (173 Ma). There are only two previously known magmatic occurrences around the Demerara Plateau. Basalts were drilled at one site on the Plateau (FG02: Fig. 2) and yielded lower Cretaceous (120–126 Ma) ages^13^. Onshore, numerous dykes are found in French Guiana and Suriname, dated at 196 Ma^30^, and were assigned to the Central Atlantic Magmatic Province (CAMP)^31^.

Basalts and rhyolites from the 60° Ridge (dredges C1 and C2) are not significantly altered and may represent primary and differentiated members of a single magmatic suite, respectively, as frequently observed in large igneous provinces (North Atlantic: Hebrides^32^, Greenland^33^; Parana-Etendeka^34^; Yemen^35^). Because of alteration, geochemical analysis of magmatic rocks from the Bastille Plateau (dredge E1) should be used with caution. It is likely that the rocks were originally mafic in composition. They share similar REE compositions with 60° Ridge samples, making a common origin possible, although they may be representative of a distinct temporal and/or source magmatic event. It is also noteworthy that all the dredged magmatic rocks have similar REE patterns (Fig. 4) and high-Ti contents (for the mafic rocks: Table S1) with the CAMP-related Guiana tholeiites^36^ and Sierra Leone intrusions^37,38^, which are more than 20 Ma older.

Unfortunately, the available seismic lines (GUYAPLAC^10^ and IGUANES^39^ cruises) do not permit a robust connection between the outcropping slopes and the SDRs identified and mapped by Reuber *et al*.^4^ and Mercier de Lépinay^3^, implying that their geometrical relationships remain unconstrained. However, SDR formation is older than the upper Jurassic, and sub-contemporaneous with the onset of oceanic spreading west of Demerara^3,4^, which should have occurred circa 170 Ma^8^. The age measured from the rhyolite (base of Middle Jurassic: 173.4 Ma) fits well with these stratigraphic constraints. We therefore propose that the dredged magmatic rocks belong to the SDR package. This interpretation brings together OIB lavas and SDRs, as commonly observed along magmatic divergent margins^40^.

The connection between a large igneous province and a hotspot track should demonstrate secular evolution and thus kinematic models should also be correlative. For example, Maia *et al*.^41^ or Le Voyer *et al*.^42^ suggest the Sierra Leone hotspot was located close to the St Peter and St Paul islets, based on Schilling *et al*.^43^ who used Duncan and Richards^44^ and Cande *et al*.^45^ to extrapolate the present-day location from the vicinity of a fixed hotspot with the mid-Atlantic ridge 75 Ma ago.

In this paper we used the kinematic reconstruction from Müller *et al*.^26^ to localize the past position of the Sierra Leone hotspot, and not as a proof of its track. However, as the uncertainties are low for recent times, and hotspot fixity is not discernable from uncertainties^46^, the recent track (<70 Ma for Müller *et al*.^26^) can be used with relative confidence. The track older than 70 Ma is mainly controlled by the correction in longitude applied in the model, explaining the large discrepancies between different models using various corrections: a fixed hotspot below the 60° Ridge at 173.4 Ma results in a present-day location in the Sierra Leone abyssal plain for Seton *et al*.^47^, or on the western edge of the Guinea Plateau for Matthews *et al*.^48^, both being more than 1000 km East or Northeast of Muller’s position (Fig. 7). Both Seton *et al*.^47^ and Matthews *et al*.^48^ reconstructions fail to explain the building of the Ceara and Sierra Leone rises with a hotspot location below Demarara at 173.4 Ma.

Therefore, Müller *et al*.^26^ kinematic reconstruction succeeds in connecting a single hotspot, namely the Sierra Leone hotspot, to the creation of both pairs of plateaus: that is, the Demerara and Guinea marginal plateaus during lower to middle Jurassic times, and Ceara and Sierra Leone oceanic plateaus during the upper Cretaceous times. Extending back in time the same kinematic model and the same assumptions of stationarity of hotspots brings the postulated Sierra Leone hotspot north of the Blake Plateau^49^, offshore South Carolina, and below the SDRs of the Carolina trough and the Blake Plateau^49,50^. In other words, the hotspot was located at the center of the Central Atlantic Magmatic Province (CAMP) 201 Ma ago, at the intersection point between the CAMP-related dykes of Africa, North and South America^51,52^. Burke and Torsvik^2^ and Ruiz-Martinez *et al*.^53^ previously noticed this coincidence between reconstructed locations of the Sierra Leone hotspot and the CAMP. Although the age of the SDRs from the Carolina trough and the Blake Plateau is debated^54^, they appear to pre-date the onset of oceanic spreading, circa 190 Ma^8^. Hence a single hotspot may have formed successive sets of SDRs during the southward propagation of the Central Atlantic, from 200–190 Ma along the east coast of North America to 180–170 Ma in Demerara, at the southernmost tip of the Central Atlantic.

However, the duration of hotspot magmatism comes into question. Some of the hotspots were proposed to be perennial since Mesozoic times: Golonka and Bocharova^55^ proposed to identify the Permo-Triassic Siberian traps with the Iceland hotspot, the Karoo-Ferrar Middle Jurassic LIP with the Bouvet hotspot, and the opening of the Central Atlantic (i.e. the CAMP) with the Sierra Leone hotspot. Here, we suggest a duration of up to 180 Ma (possibly 200 Ma) for the Sierra Leone hotspot. However, its surface expression appears to be mainly controlled by the plate tectonic setting: marginal plateaus (Demerara and Guinea) as oceanic plateaus (Ceara and Sierra Leone) formed by eruption or intrusion of large volumes of magmas when the hotspot was located below a divergent plate boundary, either during the Jurassic rifting of the Central Atlantic, or during the Cretaceous oceanic spreading in the Equatorial Atlantic. We interpret the western part of the Bathymetrists seamounts to represent the morphologic expression of the hotspot track below oceanic lithosphere in intraplate setting, with the Knipovitch seamount interpreted as the most recent volcano, expected to be 10 Ma-old.

On the contrary, the hotspot track does not clearly extend to the continental lithosphere. The Sierra Leone hotspot is expected to have stayed for a long time (from middle Jurassic to the end of Lower Cretaceous, Fig. 8B,C) below the continental margin of the West Africa, where volcanic remnants should be buried below a thick sedimentary cover. Volcanic remnants dated 104–106 Ma offshore Guinea (Los island syenites^56^ and drilled offshore trachytes^57^) may however be related to the vicinity of the hotspot (Fig. 8C).

## Conclusions

The main result of this study is the discovery of Middle Jurassic (173.4 Ma) magmatism on the northern edge of the Demerara Plateau. The magmatic suite includes mafic (basalts) and differentiated (rhyolites) rocks. Trace element patterns indicate that the basaltic rocks are similar to ocean island basalts (OIB). The magmatism is interpreted to be part of the SDRs observed in the basement of the Demerara Plateau. It supports the previous conclusions of seismic line analysis^3,4^, concluding that the Demerara Plateau formed during the Jurassic as a magmatic divergent margin at the edge of the Central Atlantic. The OIB-type and the SDRs suggest that this Jurassic magmatism is associated with a hotspot track. Kinematic reconstruction^26^ allows us to connect the Middle Jurassic creation of the Demerara and conjugated Guinea marginal plateaus, the Upper Cretaceous building of conjugated Ceara and Sierra Leone oceanic plateaus, and the Cenozoic Bathymetrists seamount chain, to a single hotspot track, namely the Sierra Leone hotspot. The last topographic remnant of the hotspot track should be the Knipovitch seamount that we think formed 10 million years ago.

## Methods

For geochemical analysis, rock samples were crushed with a stainless steel jaw crusher and then powdered in an agate swing mill. Major element concentrations were obtained using an ICP-AES Jovin Yvon Ultima 2 at University of Brest, after a HF-HNO_3_ digestion as described in Cotten *et al*.^58^. Trace element concentrations were measured with a Thermo Element2 HR-ICP-MS in Brest, after a repeated HF-HClO_4_ digestion, and HNO_3_ dilutions (see Mougel *et al*.^59^ for details). The repeated analysis of the international standards BHVO2, BCR2 and BIR1 demonstrated an external reproducibility of better than 2–10% depending on the element and concentration, with the exception of Pb (reproducibility 9–25%) (Fig. S4 and Table S1).

## Supplementary Information

Supplementary Information
