# Supplementary material for: The Jurassic magmatism of the Demerara Plateau (offshore French Guiana) as a remnant of the Sierra Leone hotspot during the Atlantic rifting
Source: Sci Rep. 2020 May 4;10:7486. doi: 10.1038/s41598-020-64333-5 (PMC7198611; doi:10.1038/s41598-020-64333-5)
Supplement: Supplementary file 1 — Supplementary Information [file 41598_2020_64333_MOESM1_ESM.docx]

Supplementary material for article

The Jurassic magmatism of the Demerara Plateau (offshore French Guiana) as a remnant of the Sierra Leone hotspot during the Atlantic rifting

**Christophe Basile^1^ (corresponding author), Igor Girault^1*^, Jean-Louis Paquette^2^, Arnaud Agranier^3^, Lies Loncke^4^, Arnauld Heuret^5^, Ewald Poetisi^6^**

^1^ Univ. Grenoble Alpes, Univ. Savoie Mont Blanc, CNRS, IRD, IFSTTAR, ISTerre, 38000 Grenoble, France. christophe.basile@univ-grenoble-alpes.fr

^2^ Université Clermont Auvergne, CNRS, IRD, OPGC, Laboratoire Magmas et Volcans, F-63000 10 Clermont-Ferrand, France. J.L.Paquette@opgc.univ-bpclermont.fr

^3^Laboratoire Géosciences Océan (UMR CNRS 6538), Université de Bretagne Occidentale & Institut Universitaire Européen de la Mer, Place Nicolas Copernic, 29280 Plouzané, France. [arnaud.agranier@univ-brest.fr](mailto:arnaud.agranier@univ-brest.fr)

^4^ Université de Perpignan, CEFREM – UMR 5110, 66860 Perpignan France. lies.loncke@univ-perp.fr

^5^ Université de Guyane, Géosciences Montpellier 97300 Cayenne, France. arnauld.heuret@univ-guyane.fr

^6^ Anton de Kom University of Suriname, Paramaribo, Suriname, SA. ewald.poetisi@uvs.edu

^*^ Present address IDEES (UMR 6266 CNRS), Université de Rouen, France. igor.girault@univ-rouen.fr

Supplementary material includes the geochemical analysis (table 1), and four figures related to geochemical analysis (S4), their interpretation (S1 and S2), and past kinematic reconstruction (S3).

**Table S1.** Chemical analysis of magmatic DRADEM samples

|  | Samples |  |  |  |  |  |  |  |  |  |  |  | standards |  |  |  |
| --- | --- | --- | --- | --- | --- | --- | --- | --- | --- | --- | --- | --- | --- | --- | --- | --- |
|  | DRA-C1-1 | DRA-C1-2 | DRA-C2-1 | DRA-E1-8c-12 | DRA-E1-9b | DRA-E1-9d | DRA-E1-1C | DRA-E1-1D | DRA-E1-2 | DRA-E1-4 | DRA-E1-5 | DRA-E1-8b-3 | BHVO2 | BHVO2 (duplicate) | BIR1 | BCR2 |
| Petrology | rhyolite | rhyolite | basalt | interm. | interm. | interm. | interm. | interm. | interm. | interm. | interm. | interm. |  |  |  |  |
| ICPOES (wt) |  |  |  |  |  |  |  |  |  |  |  |  |  |  |  |  |
| SiO2 | 73.4 | 72.3 | 47.2 | 49.2 | 45.2 | 47.7 | 47.6 | 45.6 | 47.7 | 48.1 | 45.3 | 49.1 |  |  |  |  |
| TiO2 | 0.5 | 0.5 | 3.0 | 3.4 | 2.7 | 3.4 | 3.9 | 3.5 | 3.2 | 3.9 | 3.6 | 4.1 |  |  |  |  |
| Al2O3 | 11.5 | 11.7 | 14.2 | 15.6 | 16.1 | 15.6 | 15.5 | 16.9 | 18.4 | 18.5 | 17.4 | 15.7 |  |  |  |  |
| Fe2O3 | 2.5 | 2.7 | 13.3 | 13.7 | 12.9 | 12.9 | 13.9 | 9.6 | 9.1 | 9.6 | 8.8 | 10.7 |  |  |  |  |
| MnO | 0.0 | 0.0 | 0.2 | 0.2 | 0.1 | 0.1 | 0.2 | 0.0 | 0.6 | 0.1 | 0.1 | 0.3 |  |  |  |  |
| MgO | 0.2 | 0.3 | 5.3 | 2.7 | 4.0 | 3.5 | 3.8 | 3.0 | 3.8 | 3.0 | 3.0 | 2.9 |  |  |  |  |
| CaO | 1.3 | 1.1 | 8.9 | 2.5 | 4.4 | 2.1 | 1.4 | 6.9 | 4.0 | 4.9 | 7.4 | 3.5 |  |  |  |  |
| Na2O | 3.1 | 3.2 | 3.1 | 5.5 | 3.8 | 3.9 | 5.0 | 2.6 | 3.0 | 3.0 | 2.8 | 5.0 |  |  |  |  |
| K2O | 4.5 | 4.6 | 1.0 | 2.1 | 2.3 | 3.7 | 1.3 | 3.4 | 3.2 | 2.6 | 3.1 | 2.0 |  |  |  |  |
| P2O5 | 0.1 | 0.1 | 0.4 | 0.4 | 1.5 | 0.5 | 0.5 | 0.4 | 0.4 | 0.4 | 0.4 | 0.5 |  |  |  |  |
| LOI | 0.3 | 0.3 | 2.1 | 3.2 | 6.0 | 5.2 | 5.8 | 7.1 | 5.1 | 4.8 | 7.1 | 4.7 |  |  |  |  |
| Total | 97.5 | 96.7 | 98.8 | 98.6 | 99.0 | 98.6 | 99.0 | 98.9 | 98.7 | 98.7 | 99.0 | 98.4 |  |  |  |  |
|  | 7.5 | 7.8 | 4.1 | 7.6 | 6.1 | 7.6 | 6.3 | 6.0 | 6.2 | 5.6 | 5.9 | 7.0 |  |  |  |  |
| HR-ICP-MS (ppm) |  |  |  |  |  |  |  |  |  |  |  |  |  |  |  |  |
| Y | 57.9 | 16.2 | 54 | 15.1 | 49.6 | 38.1 | 17.2 | 15.7 | 15.4 | 4.64 | 15.2 | 23.2 | 30.3 | 29.1 | 16.5 | 36.6 |
| La | 52.6 | 11 | 32.7 | 5.76 | 25.1 | 15.4 | 7.94 | 6.68 | 10.2 | 2.68 | 8.81 | 11 | 16.9 | 16.3 | 0.641 | 23.7 |
| Ce | 145 | 37.6 | 75.3 | 27.9 | 38.1 | 33.9 | 31 | 31.7 | 28.1 | 19.4 | 28.1 | 33.1 | 36.1 | 38.9 | 1.96 | 51 |
| Pr | 14.5 | 3.69 | 9.88 | 1.97 | 5.75 | 5.19 | 2.62 | 2.49 | 3.29 | 1.09 | 2.99 | 3.73 | 5.36 | 5.13 | 0.357 | 6.48 |
| Nd | 56.7 | 15.4 | 42.7 | 8.81 | 25.6 | 23.6 | 12 | 11.9 | 14.7 | 5.21 | 13.8 | 16.9 | 24.2 | 23.5 | 2.27 | 27.2 |
| Sm | 11.8 | 3.76 | 9.97 | 2.36 | 6.13 | 6.39 | 3.06 | 3.41 | 3.59 | 1.42 | 3.49 | 4.35 | 6.04 | 5.78 | 1.07 | 6.18 |
| Eu | 2.31 | 0.837 | 2.67 | 0.776 | 1.84 | 1.56 | 0.593 | 1.33 | 1.17 | 0.546 | 1.35 | 1.14 | 1.99 | 1.9 | 0.472 | 1.85 |
| Tb | 1.79 | 0.652 | 1.61 | 0.415 | 1.12 | 1.13 | 0.508 | 0.582 | 0.548 | 0.209 | 0.555 | 0.7 | 0.958 | 0.918 | 0.34 | 1 |
| Ho | 2.05 | 0.824 | 1.83 | 0.497 | 1.39 | 1.33 | 0.553 | 0.603 | 0.565 | 0.204 | 0.56 | 0.762 | 0.981 | 0.924 | 0.529 | 1.19 |
| Er | 5.81 | 2.41 | 5.08 | 1.44 | 3.95 | 3.73 | 1.56 | 1.56 | 1.48 | 0.514 | 1.44 | 2.12 | 2.55 | 2.39 | 1.64 | 3.4 |
| Tm | 0.867 | 0.365 | 0.729 | 0.206 | 0.569 | 0.537 | 0.212 | 0.204 | 0.198 | 0.0635 | 0.189 | 0.291 | 0.341 | 0.321 | 0.245 | 0.494 |
| Lu | 0.763 | 0.337 | 0.65 | 0.189 | 0.519 | 0.466 | 0.169 | 0.152 | 0.154 | 0.0463 | 0.136 | 0.224 | 0.278 | 0.261 | 0.233 | 0.463 |
| Li | 0.478 | 0.298 | 9.34 | 16.2 | 41.3 | 45.7 | 47.2 | 33.4 | 52.8 | 25.1 | 33.9 | 25.4 | 4.85 | 4.6 | 2.97 | 9.22 |
| Be | 4.96 | 4.39 | 2.14 | 1.23 | 0.981 | 0.672 | 0.629 | 0.88 | 1.02 | 0.898 | 0.779 | 0.868 | 1.16 | 1.04 | 0.0941 | 2.59 |
| Zr | 365 | 313 | 282 | 266 | 216 | 246 | 272 | 218 | 208 | 214 | 218 | 255 | 180 | 173 | 14.9 | 180 |
| Nb | 72.4 | 70.6 | 35.1 | 25.9 | 19.7 | 20.4 | 27.7 | 19.5 | 19.2 | 22.5 | 20.6 | 23.8 | 19.1 | 18.6 | 0.561 | 12.7 |
| Cs | 0.291 | 0.323 | 0.456 | 0.0449 | 0.375 | 0.211 | 0.161 | 0.23 | 0.347 | 0.0893 | 0.275 | 0.0759 | 0.104 | 0.101 | 0.0038 | 1.1 |
| Hf | 10.5 | 8.84 | 7.81 | 6.24 | 4.97 | 5.42 | 6.62 | 5.34 | 5.01 | 5.33 | 5.27 | 5.96 | 4.33 | 4.07 | 0.561 | 4.57 |
| Ta | 4.76 | 4.53 | 2.14 | 1.59 | 1.16 | 1.21 | 1.63 | 1.22 | 1.13 | 1.32 | 1.22 | 1.42 | 1.14 | 1.07 | 0.0438 | 0.726 |
| Pb | 9.7 | 8.91 | 3.55 | 2.43 | 2.52 | 13.4 | 2.05 | 1.81 | 2.41 | 2.21 | 1.86 | 2.29 | 1.91 | 3.4 | 3.01 | 9.96 |
| Th | 7.52 | 2.9 | 2.81 | 1.52 | 1.34 | 1.14 | 1.2 | 0.917 | 0.911 | 0.365 | 0.82 | 1.11 | 1.13 | 1.05 | 0.0259 | 5.09 |
| U | 2.42 | 1.32 | 0.729 | 1.06 | 1.33 | 1.31 | 2.15 | 1.44 | 3.07 | 1.37 | 1.43 | 2.67 | 0.409 | 0.393 | 0.0184 | 1.55 |
| Sc | 4.61 | 1.65 | 37.7 | 10.8 | 39.5 | 36.9 | 27.7 | 36.8 | 25.6 | 8.16 | 26.3 | 22.3 | 36.4 | 34.6 | 42.4 | 35.9 |
| Ti | 2896 | 2680 | 18340 | 19240 | 15360 | 18690 | 22610 | 18920 | 18680 | 21310 | 20070 | 23250 | 16250 | 15470 | 5355 | 13360 |
| V | 15.5 | 14.3 | 415 | 497 | 342 | 469 | 586 | 489 | 399 | 538 | 431 | 536 | 330 | 306 | 306 | 418 |
| Cr | 10.7 | 17.9 | 137 | 76.8 | 80.4 | 192 | 84.9 | 119 | 151 | 131 | 128 | 135 | 383 | 348 | 356 | 20.4 |
| Co | 1.13 | 1.37 | 39.5 | 37.4 | 47.5 | 42.6 | 44.3 | 42.7 | 57.2 | 38 | 36.4 | 48.6 | 47.8 | 46.6 | 50 | 37 |
| Ni | 1.18 | 1.6 | 61.3 | 52.6 | 135 | 142 | 133 | 74.9 | 251 | 67 | 67.8 | 171 | 118 | 115 | 164 | 11.8 |
| Cu | 5.84 | 7.7 | 149 | 83.9 | 320 | 618 | 118 | 214 | 143 | 338 | 199 | 211 | 138 | 138 | 122 | 26.4 |
| Ga | 19.9 | 15.5 | 23.2 | 23.7 | 22.8 | 20.1 | 23.6 | 24.3 | 25.7 | 23.7 | 24.5 | 23.8 | 22.3 | 21.6 | 15.5 | 22.6 |
| Ge | 1.44 | 0.735 | 1.95 | 1.89 | 2.33 | 2.59 | 1.76 | 1.58 | 1.81 | 1.33 | 1.49 | 2.1 | 1.84 | 1.85 | 1.23 | 1.8 |
| Rb | 91.4 | 60.7 | 29.7 | 14.8 | 25.6 | 49.8 | 15.8 | 20.6 | 22.4 | 18.3 | 17.6 | 10.5 | 10.8 | 10.6 | 0.316 | 48 |
| Sr | 82 | 40.1 | 291 | 75.8 | 338 | 121 | 188 | 158 | 225 | 122 | 199 | 30 | 412 | 388 | 99.5 | 335 |
| Ba | 836 | 694 | 345 | 285 | 488 | 502 | 270 | 154 | 179 | 166 | 156 | 35.1 | 130 | 121 | 6.72 | 665 |
| Eu | 2.3 | 0.903 | 2.65 | 0.81 | 1.9 | 1.63 | 0.623 | 1.32 | 1.19 | 0.57 | 1.36 | 1.13 | 1.97 | 1.91 | 0.486 | 1.94 |
| Gd | 11.1 | 3.75 | 10.2 | 2.54 | 6.94 | 6.91 | 3.24 | 3.65 | 3.59 | 1.39 | 3.59 | 4.51 | 6.21 | 5.99 | 1.8 | 6.45 |
| Dy | 10.4 | 4.03 | 9.31 | 2.5 | 6.68 | 6.74 | 2.88 | 3.26 | 3 | 1.11 | 3.05 | 4 | 5.37 | 5.04 | 2.36 | 6.03 |
| Yb | 5.25 | 2.34 | 4.49 | 1.31 | 3.5 | 3.21 | 1.22 | 1.17 | 1.1 | 0.358 | 1.03 | 1.69 | 1.99 | 1.9 | 1.54 | 3.08 |
| Tl | 0.915 | 1.02 | 0.343 | 2.38 | 2.66 | 13.1 | 1.45 | 1.45 | 8.32 | 0.863 | 1.06 | 5.49 | 0.465 | 0.231 | 0.0302 | 0.924 |

**Table S2.** Zircon U-Pb data from DRA-C1 rhyolite, obtained by in situ Laser Ablation ICP-MS

|  |  |  |  |  |  | **2 σ error** |  | **2 σ error** |  | **Age (Ma)** | **2 σ error** |
| --- | --- | --- | --- | --- | --- | --- | --- | --- | --- | --- | --- |
| **Analysis** | **Pb ppm*^1^*** | **Th ppm***^1^* | **U ppm***^1^* | **Th/U** | **^207^Pb/^235^U***^2^* | **^207^Pb/^235^U** | **^206^Pb/^238^U***^2^* | **^206^Pb/^238^U** | **Rho** | **^206^Pb/^238^U** | **^206^Pb/^238^U** |
| Zr1/1 | 23 | 391 | 804 | 0.49 | 0.1915 | 0.0061 | 0.02713 | 0.00075 | 0.87 | 172.5 | 4.7 |
| Zr1/2 | 37 | 978 | 1174 | 0.83 | 0.1935 | 0.0060 | 0.02776 | 0.00075 | 0.87 | 176.5 | 4.8 |
| Zr1/3 | 32 | 768 | 1048 | 0.73 | 0.2041 | 0.0064 | 0.02741 | 0.00075 | 0.87 | 174.3 | 4.7 |
| Zr1/4 | 42 | 886 | 1389 | 0.64 | 0.1965 | 0.0063 | 0.02744 | 0.00075 | 0.86 | 174.5 | 4.7 |
| Zr1/5 | 34 | 724 | 1155 | 0.63 | 0.1984 | 0.0066 | 0.02738 | 0.00075 | 0.82 | 174.1 | 4.7 |
| Zr2/1 | 5.3 | 185 | 154 | 1.20 | 0.1999 | 0.0113 | 0.02747 | 0.00081 | 0.52 | 174.7 | 5.0 |
| Zr2/2 | 3.7 | 127 | 108 | 1.18 | 0.1937 | 0.0120 | 0.02732 | 0.00081 | 0.48 | 173.8 | 5.1 |
| Zr3/1 | 2.1 | 59 | 50 | 1.19 | 0.4865 | 0.0383 | 0.02902 | 0.00108 | 0.47 | 184.4 | 6.7 |
| Zr3/2 | 1.3 | 30 | 32 | 0.94 | 0.4527 | 0.0472 | 0.02952 | 0.00123 | 0.40 | 187.5 | 7.7 |
| Zr5/1 | 5.3 | 125 | 159 | 0.79 | 0.2464 | 0.0196 | 0.02789 | 0.00093 | 0.42 | 177.3 | 5.8 |
| Zr5/2 | 13 | 479 | 355 | 1.35 | 0.2662 | 0.0115 | 0.02724 | 0.00078 | 0.66 | 173.2 | 4.9 |
| Zr7/1 | 1.8 | 41 | 60 | 0.69 | 0.1961 | 0.0144 | 0.02702 | 0.00081 | 0.41 | 171.9 | 5.1 |
| Zr7/2 | 1.7 | 33 | 51 | 0.65 | 0.3266 | 0.0200 | 0.02812 | 0.00087 | 0.51 | 178.7 | 5.4 |
| Zr7/3 | 2.1 | 53 | 67 | 0.80 | 0.1920 | 0.0143 | 0.02718 | 0.00081 | 0.40 | 172.9 | 5.2 |
| ***^1^***: concentration uncertainty c.20% | | | |  |  |  |  |  |  |  |  |
| ***^2^***: data not corrected for common-Pb | | | |  |  |  |  |  |  |  |  |

**Figure S1.** SiO_2_ vs Loss of ignition (L.O.I.). A: intermediate samples (orange squares) and the basaltic sample DRA C2-1 (dark gray square). B: intermediate samples (orange squares), the basaltic sample DRA C2-1 (dark gray square) and the rhyolitic samples DRA C1-1 and DRA C1-2. Note the distinct correlation between SiO_2_ and L.O.I. for samples of intermediate -compostions, which is characteristic of a SiO_2_ leaching induced by seawater weathering.


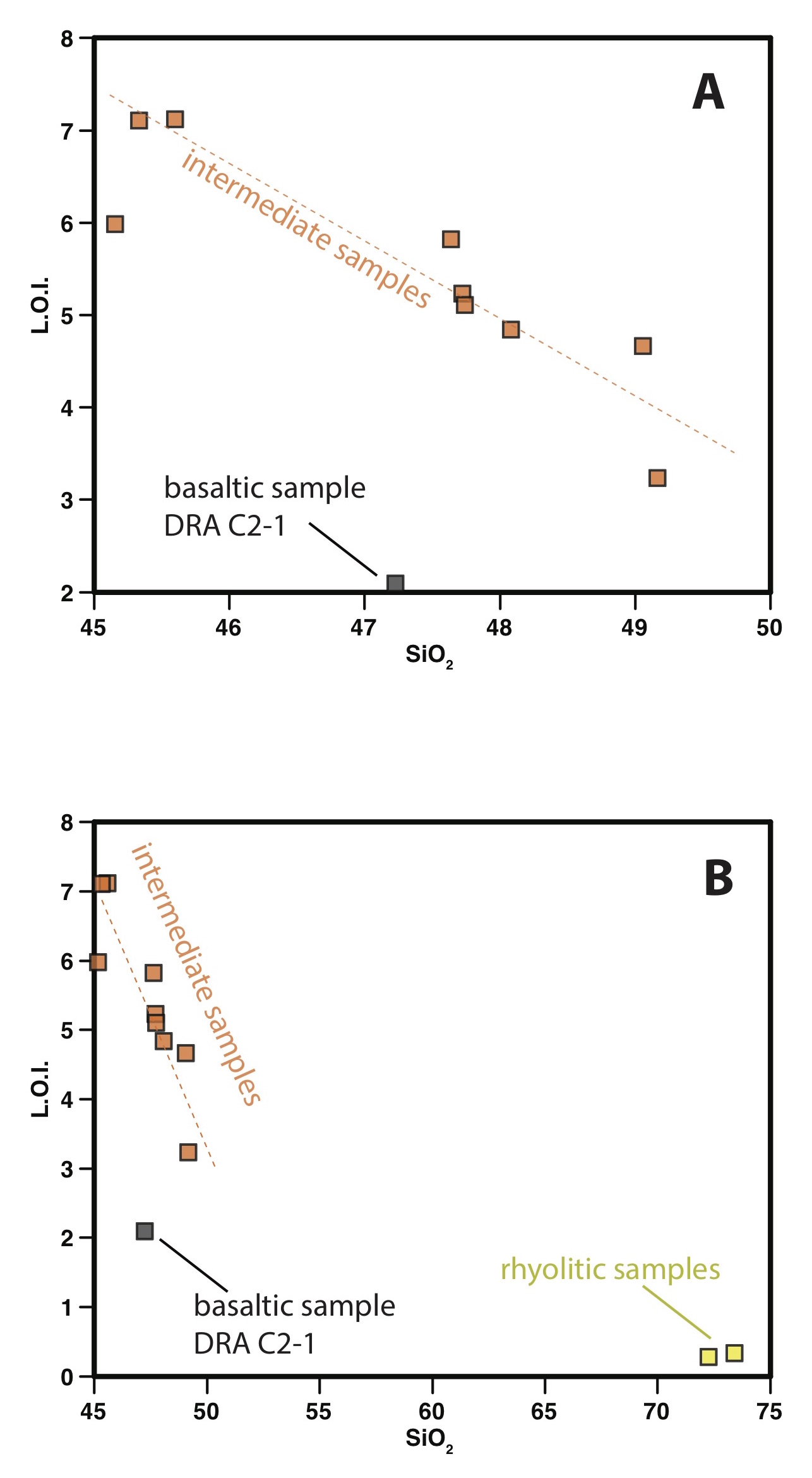


**Figure S2.** Ce/Ce* vs Nb/La in the basatic, intermediate and rhyolitic samples of this study. Ce* is estimated based on the averaged concentrations of La and Pr, normalized to the primitive mantle^58^. The apparent correlation between the rare earth element Ce and the high field strength element Nb, both non- fluid -mobile-elements under oxydative conditions (Ce_4_^+^) supports the possiblity of an apparent enrichment in these elements, due to the selective leaching of other rare earth elements. Nevertheless, the extent of the HFSE positive anomaly being approximately three times higher than Ce anomalies, it is likely that a pristine (i.e.: magmatic) enrichment in these elements was present before alteration.


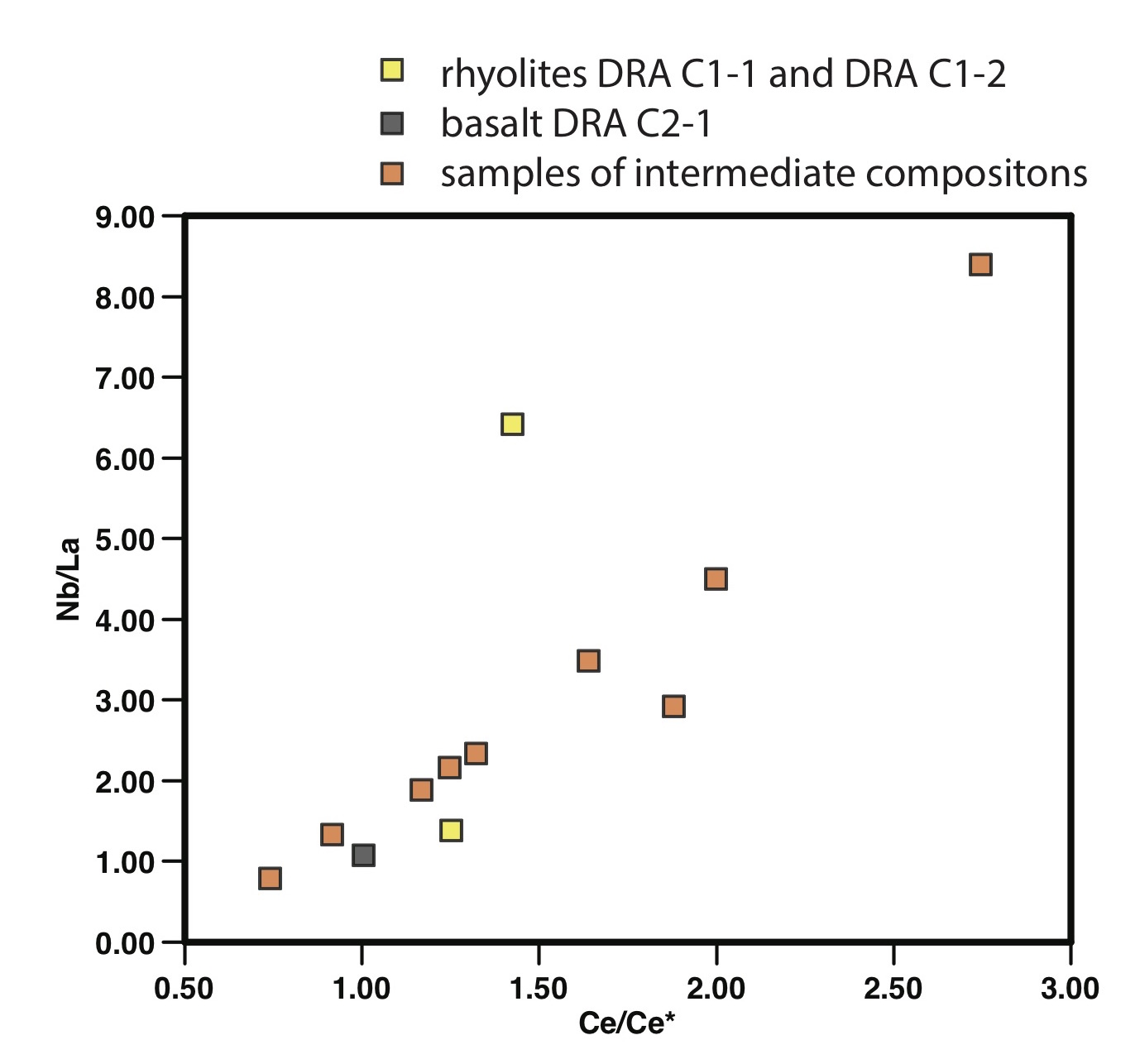


Figure S3. Position of the postulated Sierra Leone hotspot (red star) at 201 Ma, using the kinematic model of Müller et al^26^. The dashed contour indicates the position of the Blake Plateau by reference to North America. The Blake Plateau formed during the Lower Jurassic formation of the Central Atlantic^49^. Drawn from results using GPlates 2.0.0.

Figure S4. Measured international standards BHVO2, BIR1 and BCR2 compared to published referenced values^61, 62, 63^. Normalization values (primitive Mantle) are those of McDonough and Sun^58^. Measured and referenced values are similar within (2-10%).

**
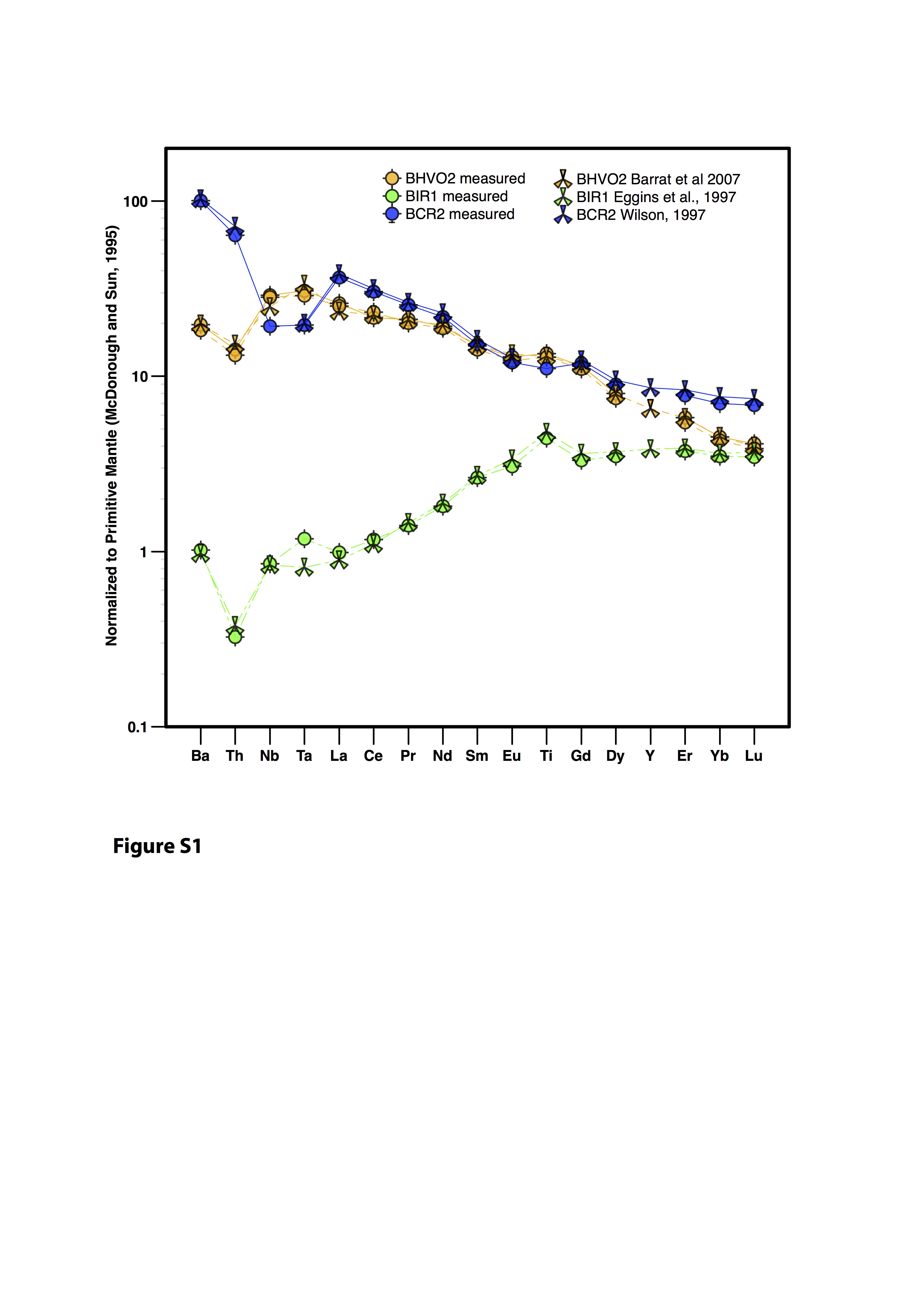
**
